# Loss of cerebellar function selectively affects intrinsic rhythmicity of eupneic breathing

**DOI:** 10.1242/bio.048785

**Published:** 2020-04-28

**Authors:** Yu Liu, Shuhua Qi, Fridtjof Thomas, Brittany L. Correia, Angela P. Taylor, Roy V. Sillitoe, Detlef H. Heck

**Affiliations:** 1 Department of Anatomy and Neurobiology, College of Medicine, University of Tennessee Health Science Center, Memphis, TN 38163, USA; 2 Division of Biostatistics, Department of Preventive Medicine, College of Medicine, University of Tennessee Health Science Center, Memphis, TN 38163, USA; 3 Department of Pathology and Immunology, Baylor College of Medicine, Houston, TX 77030, USA; Jan and Dan Duncan Neurological Research Institute, Texas Children's Hospital, Houston, TX 77030, USA

**Keywords:** Respiration, Motor skills disorder, Ataxia, Arrhythmia, Cerebellum

## Abstract

Respiration is controlled by central pattern generating circuits in the brain stem, whose activity can be modulated by inputs from other brain areas to adapt respiration to autonomic and behavioral demands. The cerebellum is known to be part of the neuronal circuitry activated during respiratory challenges, such as hunger for air, but has not been found to be involved in the control of spontaneous, unobstructed breathing (eupnea). Here we applied a measure of intrinsic rhythmicity, the CV2, which evaluates the similarity of subsequent intervals and is thus sensitive to changes in rhythmicity at the temporal resolution of individual respiratory intervals. The variability of intrinsic respiratory rhythmicity was reduced in a mouse model of cerebellar ataxia compared to their healthy littermates. Irrespective of that difference, the average respiratory rate and the average coefficient of variation (CV) were comparable between healthy and ataxic mice. We argue that these findings are consistent with a proposed role of the cerebellum in modulating the duration of individual respiratory intervals, which could serve the purpose of coordinating respiration with other rhythmic orofacial movements, such as fluid licking and swallowing.

## Abstract (precis)

**Summary:** The cerebellum was believed to have no role in controlling eupneic respiration. Our findings suggest a cerebellar involvement in the coordination of breathing with other orofacial movements, such as swallowing.

## INTRODUCTION

The cerebellum has extensive reciprocal connections with the brain stem and cerebellar neuropathology is known to affect brain stem controlled processes, such as cardiovascular and respiratory function (Harper et al., 1998; Paton et al., 1991; Rector et al., 2006). The involvement of the cerebellum in respiration seems to be central to respiratory challenges, such as hypoxia or hypercapnia (Macey et al., 2005; Parsons et al., 2001). Neurons in the medial cerebellar nuclei project to brain stem areas containing the respiratory pattern generating circuits (Lu et al., 2013) and neuronal activity in the medial cerebellar nuclei is entrained by the respiratory rhythm (Lu et al., 2013; Xu and Frazier, 2002). Investigations into eupneic respiration, however, failed to implicate the cerebellum in animal models (Moruzzi, 1940; Xu and Frazier, 2002; Xu et al., 1995) or humans (Ebert et al., 1995). Previously published findings suggested that ablation of the fastigial nucleus altered respiratory responses to hypercapnia but did not alter eupneic breathing (Xu and Frazier, 2002). However, a recent study conducted in juvenile mice reported the possibility of only a modest influence of cerebellar cortical function during respiration as determined by a measure of breathing regularity called inter-breath interval (van der Heijden and Zoghbi, 2018). Collectively, these previous studies have been complicated by the use of anaesthetized conditions or analyses conducted before postnatal day 30, an age prior to which functional cerebellar circuits have yet to reach maturity (Arancillo et al., 2015). We therefore sought to test the role of the mature cerebellum in eupneic breathing, i.e. unobstructed breathing of normoxic air, in awake conditions using multiple physiological measures of system rhythmicity.

To quantitatively address this problem, we compared the average respiratory rate, coefficient of variation (CV), of the respiratory rhythm and the intrinsic rhythmicity of respiration (CV2) (Holt et al., 1996) of eupneic breathing in healthy mice (controls, CT) and in adult mice with cerebellar ataxia. Cerebellar ataxia was induced using the Cre/LoxP genetic approach to selectively block Purkinje cell GABAergic neurotransmission (*L7^Cre^;Vgat^flox/flox^*), thereby functionally disconnecting the cerebellar cortex from the cerebellar nuclei (White et al., 2014). The gross anatomical features of the cerebellum are preserved in *L7^Cre^;Vgat^flox/flox^* (MU) mice. The loss of Purkinje cell synaptic transmission causes expected motor deficits that are typical for cerebellar neuropathology, such as a severely ataxic gait (White et al., 2014). Here, we measured spontaneous respiratory behavior in MU and CT mice over a 30-min period in a plethysmograph.

Cerebellar ataxia did not affect the average respiratory rate or the CV of the respiratory rhythm. However, compared to their CT littermates, MU mice showed increased intrinsic rhythmicity, as measured by the CV2. The CV2 evaluates the similarity of pairs of intervals, and is thus sensitive to brief changes in rhythmicity at a temporal resolution of individual interval durations. The CV2 provides a measure of intrinsic rhythmicity in the sense that this measure is sensitive to short periods of highly regular breathing, but less sensitive to slow rate variations than the CV. Based on our results and findings from previously published studies, we propose a role for the cerebellum in the coordination of multiple rhythmic orofacial movements, such as coordinating respiration with swallowing.

## RESULTS

**Fig. 1.**
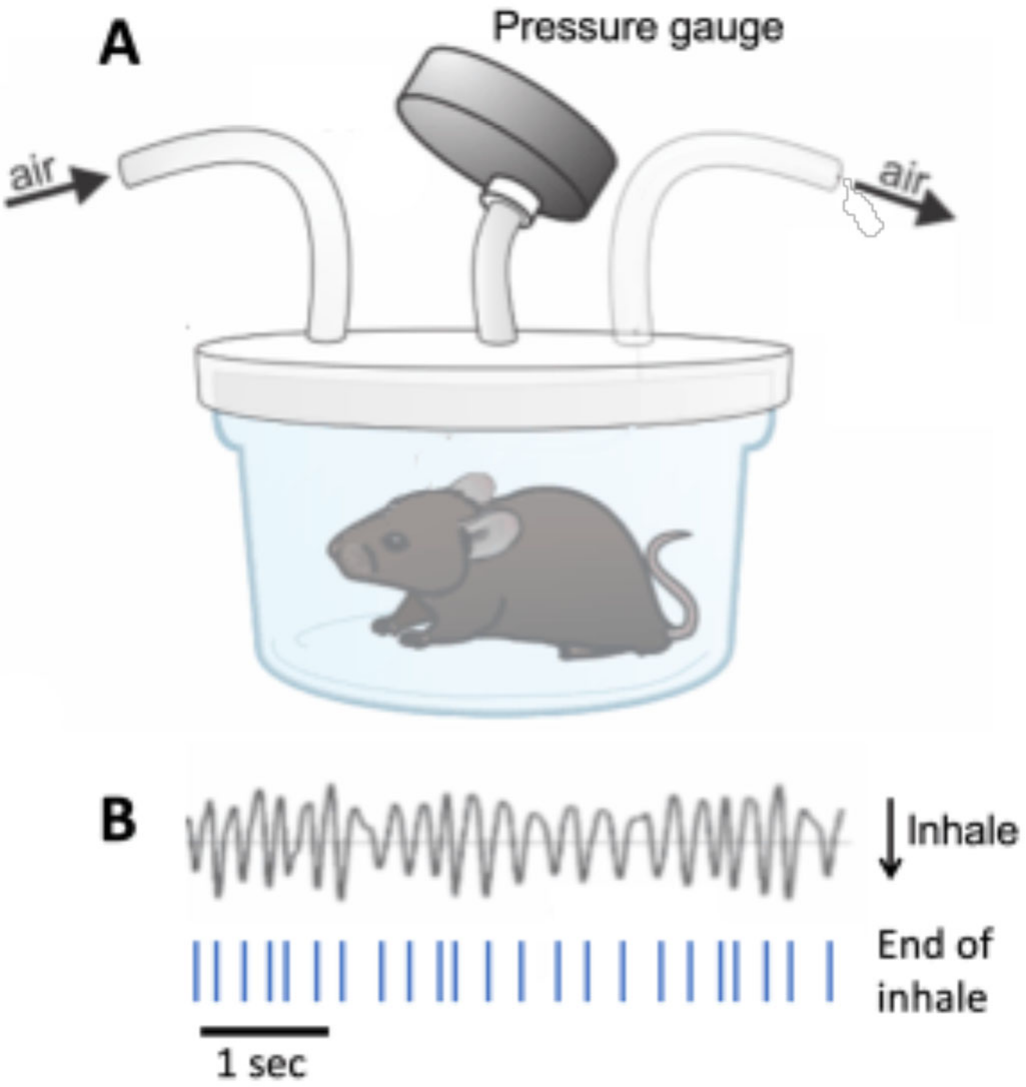
**Mouse respiratory behavior was measured using a custom-made plethysmograph chamber.** (A) Pressure inside the chamber was measured with a pressure transducer (Validyne Engineering, USA). Inhalation movements increase the pressure inside the chamber, resulting in decreases in voltage. (B) Raw voltage output from the pressure transducer reflecting respiration-related pressure changes. Inhalation-related increases in pressure are reflected as decreases in voltage. Troughs in the voltage signal reflect the end of inhale times, which were marked and used as temporal alignments for further analysis of the respiratory rhythm.

To measure spontaneous respiratory behavior, mice were placed in a plethysmograph chamber where they could move freely (Fig. 1). Spontaneous respiration was monitored for 30 min and compared between MU mice and their CT littermates (Table 1). Male and female mice of both genotypes were divided into two age groups, 2–3 or 5–7 months old (Table 1), corresponding approximately to the transition from adolescents/young adults to mature adults in human development (Flurkey et al., 2007).

**Table 1.**
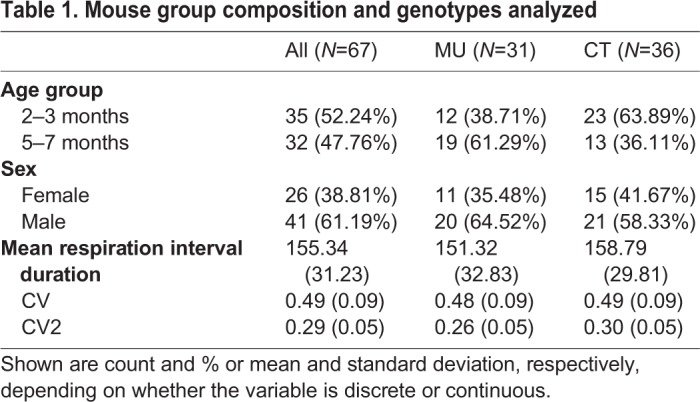
**Mouse group composition and genotypes analyzed**

Respiratory activity in the plethysmograph chamber causes rhythmic pressure changes, which were measured using a pressure transducer (Fig. 1A). Rhythmic decreases in voltage output from the transducer corresponded to inhalation movements, and the times of voltage trough minima were marked as ‘end-of-inhalation’ times (Fig. 1B). All further analysis was based on these temporal markers.

**Fig. 2.**
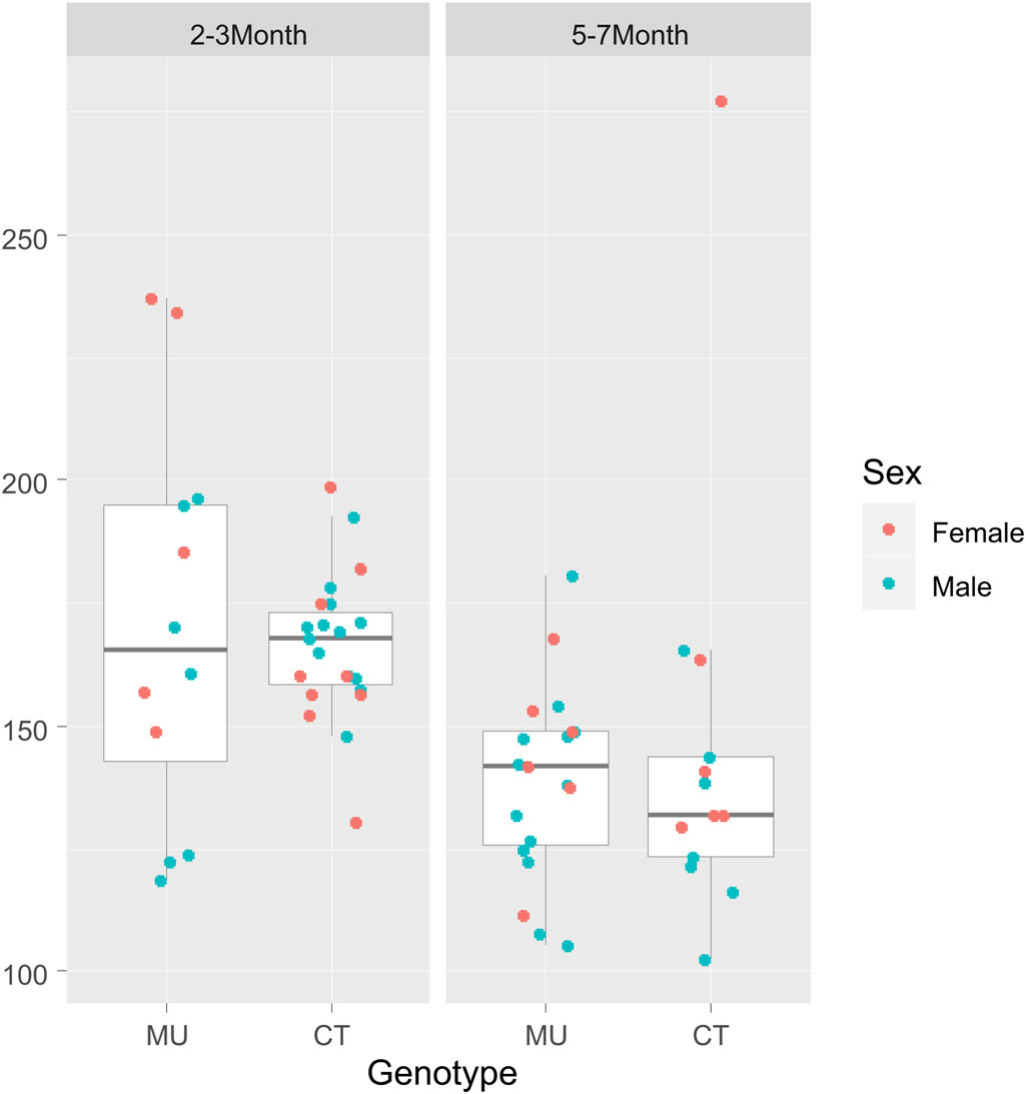
**Comparison of mean respiratory interval durations.** Mean interval duration is lower in older mice (right panel; *P*<0.001), but does not differ between MU and CT mice within each age-group (left and right box within each panel; *P*=0.99, adjusted for age and sex). There seems to be a systematic albeit modest difference by sex (*P*=0.048, adjusted for age).

The average respiratory rate was evaluated by calculating the average duration of inter-inhalation intervals. Comparison of mean interval durations between MU mice and CT littermates within age groups revealed no difference between groups (*P*=0.99, adjusted for sex and age; Fig. 2 – see Materials and Methods/statistical analysis for details). Older mice, however, had shorter average interval durations than the younger mice (*P*<0.001; adjusted for sex), consistent with a known age-related increase in respiratory rate (Rodríguez-Molinero et al., 2013). Comparison of respiratory rate by sex showed a systematic, albeit not very pronounced, difference (*P*=0.048, adjusted for age), with female mice having higher respiratory rates than males.

**Fig. 3.**
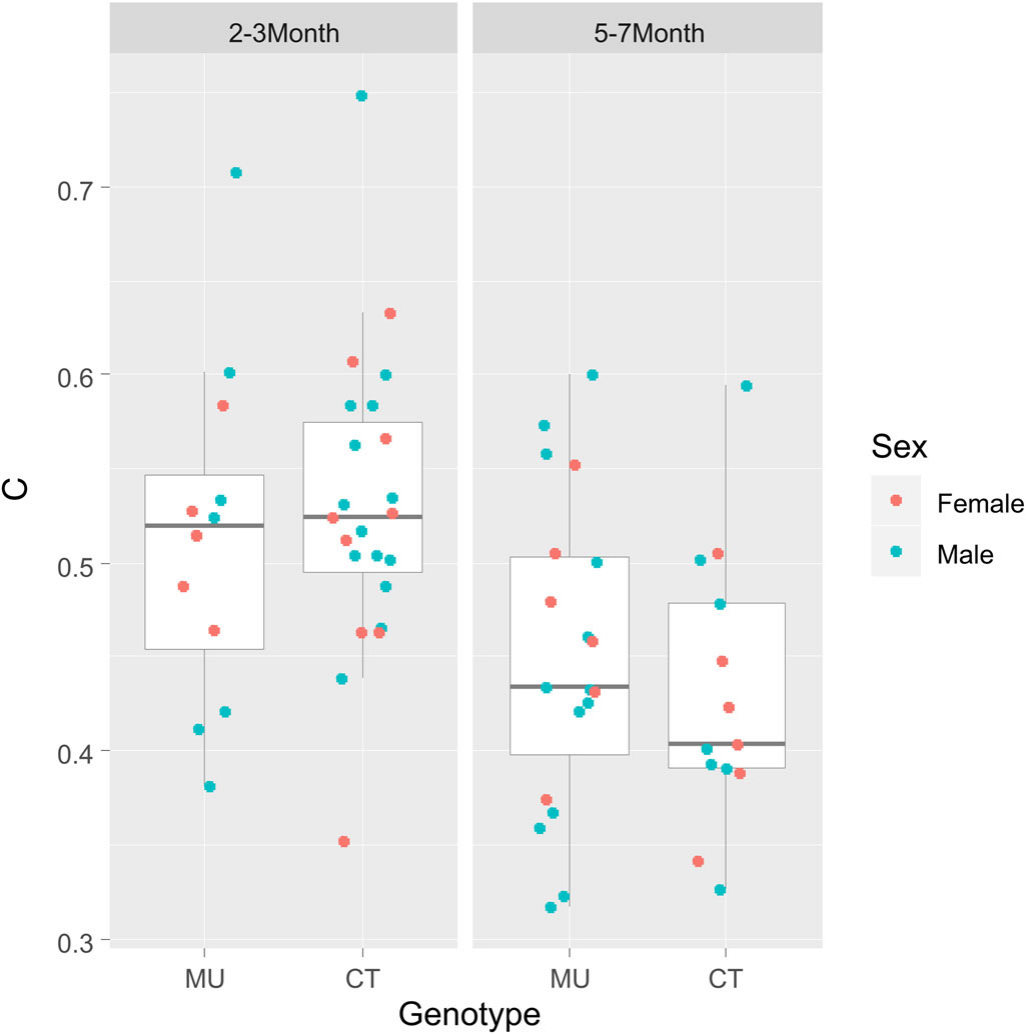
**Comparison of the coefficient of variation (CV) of the respiratory interval distribution.** The CV is lower in older mice (left versus right panel; *P*<0.001) but does not differ between MU and CT mice within each age-group (left and right box within each panel; *P*=0.95). No systematic differences in the CV were detected by sex (*P*=0.70).

The overall variability of the respiratory rhythm, quantified as the coefficient of variation of the inter-inhalation interval distribution (CV=standard deviation of interval distribution/mean of interval duration) was, like the mean interval duration, dependent on age (*P*<0.001, with lower values for younger mice), but not on sex (*P*=0.70). Also in analogy to the mean interval duration above, we could not find a difference between MU mice and their CT littermates with respect to CV (*P*=0.95, adjusted for age) (Fig. 3).

The CV measures variability based on the distribution of intervals across the entire observation time. Brief but reoccurring changes in rhythmicity cause no change in the CV, as long as the overall interval distribution remains the same or similar. In 1996, Holt et al. introduced a novel measure of variability, the CV2 (see Materials and Methods), specifically designed to detect brief, reoccurring changes in rhythmicity, which they called ‘intrinsic rhythmicity’ (Holt et al., 1996).

**Fig. 4.**
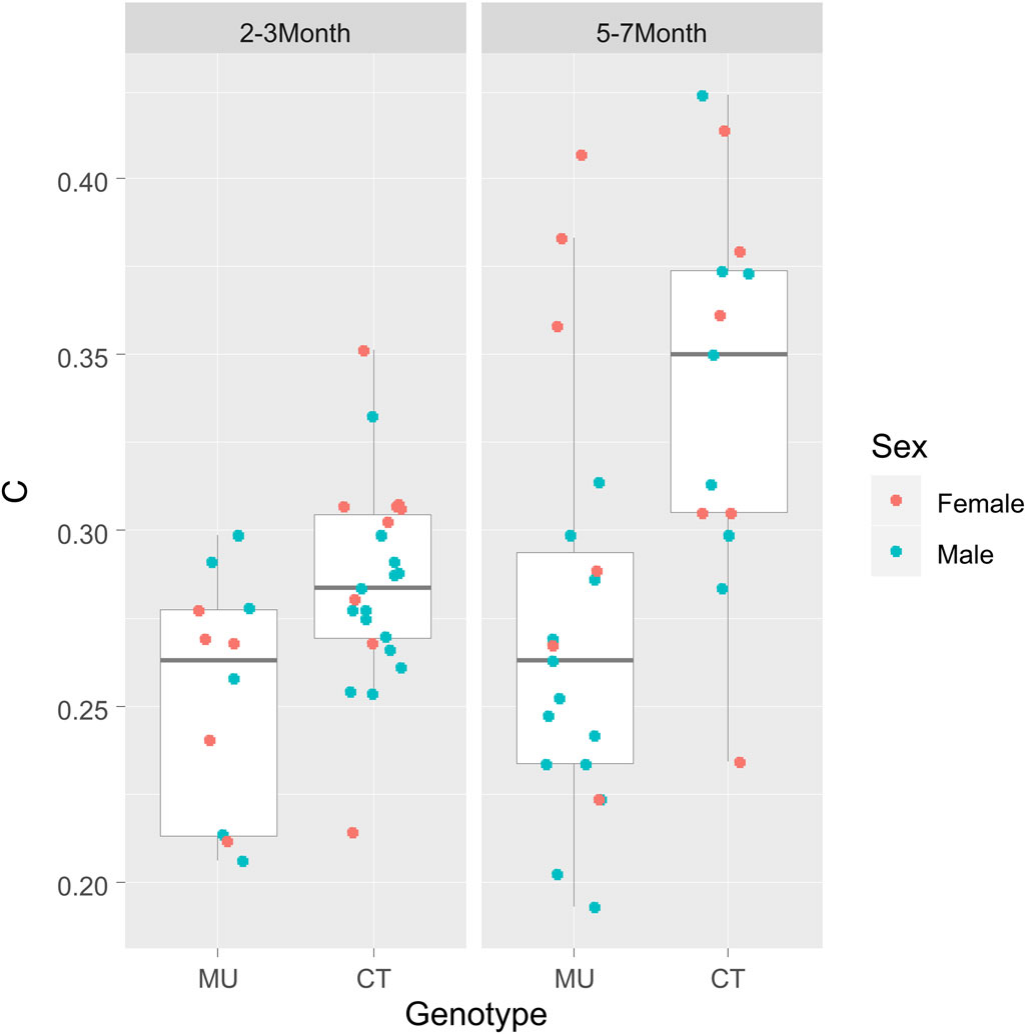
**Comparison of the CV2 (intrinsic rhythmicity) of respiratory behavior.** The CV2 is lower in the MU mice compared to their CT littermates (left versus right box in each panel; *P*<0.001) when adjusting for age (*P*<0.001) and sex (*P*=0.048). In addition, the CV2 was significantly higher in older compared to younger CT mice (*P*<0.001 in the CT subgroup), but possibly remained unchanged across age in the MU mice (*P*=0.16 in the MU subgroup). However, in the combined data, the interaction effect between age and genotype is not statistically significant (*P*=0.19); something that would be expected if the age-CV2 relationship holds for only one of the two genotypes.

**Fig. 5.**
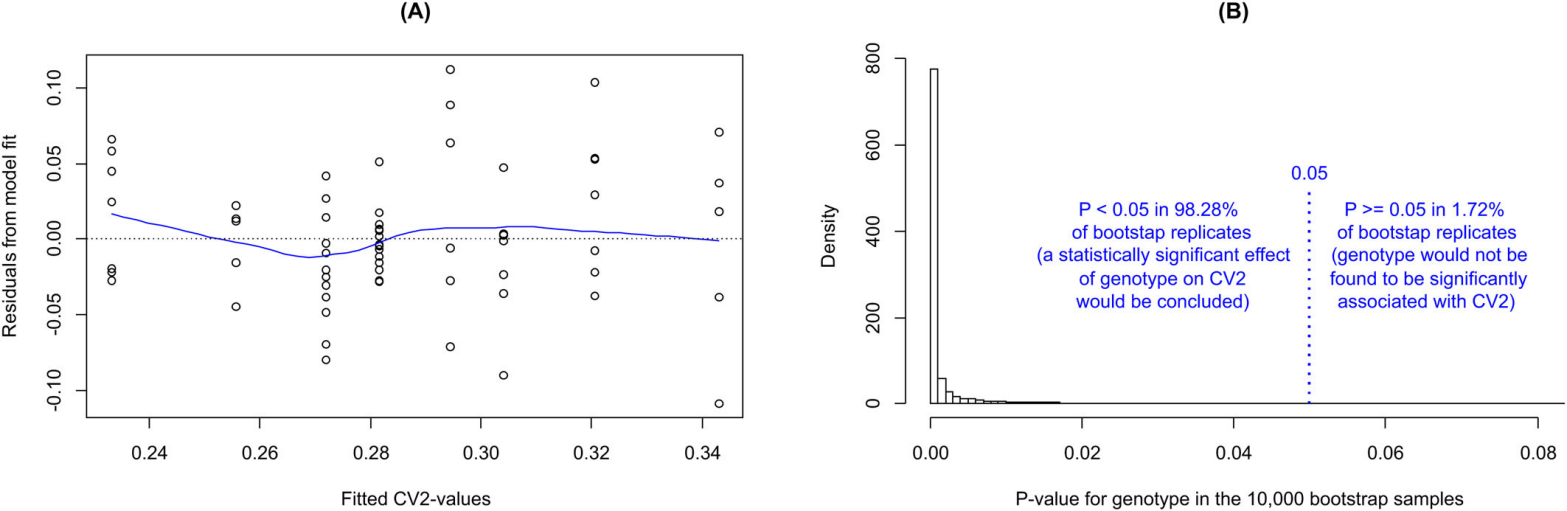
**Model fit assessment and robustness of finding.** (A) Our model specifying CV2 as a function of sex, age and genotype fits the observed data well. The blue line shows a LOESS-smoother for visual guidance: A good model fit requires that the residuals (y-axis) are independent of the CV2-values (x-axis) and generally lack ‘pattern’, which is the case here. The grouping of points into eight vertical bands is a consequence of the model being restricted to include only sex, age (two groups) and genotype, resulting in 2×2×2=8 possible combinations of covariate-values and, therefore, only eight distinct predicted (fitted) CV2 values. (B) A statistically significant association of genotype and CV2 is present in essentially all of the 10,000 bootstrap replications (98.28%) and is a robust feature of the data. Each bootstrap replication is based on re-sampling with replacement from the truly observed data with identical number of animals (but some possibly being included in the data several times due to the sampling with replacement approach while some will be dropped). The generally small *P*-values resulting from each bootstrap replication indicate that the statistical significance is not driven by a few extreme observations, but is a general feature of this data set.

Comparison of the CV2 of respiratory behavior in CT and MU mice revealed a lower CV2 in MU mice compared to the CT littermates (*P*<0.001), when adjusting for age (*P*<0.001) and sex (*P*=0.048) (Fig. 4). While the CV2 increased with age in the control mice (*P*<0.001), it did not change substantially with age in MU mice (*P*=0.16) (Fig. 4). Irrespectively, in the combined data, the interaction effect between age and genotype was not significant (*P*=0.27); something that would be expected if the age–CV2 relationship holds for only one of the two genotypes. We verified adequate fit of our model by investigating the model residuals (Fig. 5A) and confirmed by a bootstrap approach that the statistical significance of the association of CV2 and genotype is a general and robust feature of this data set (Fig. 5B) and is not driven by a few ‘extreme’ observations only.

**Fig. 6.**
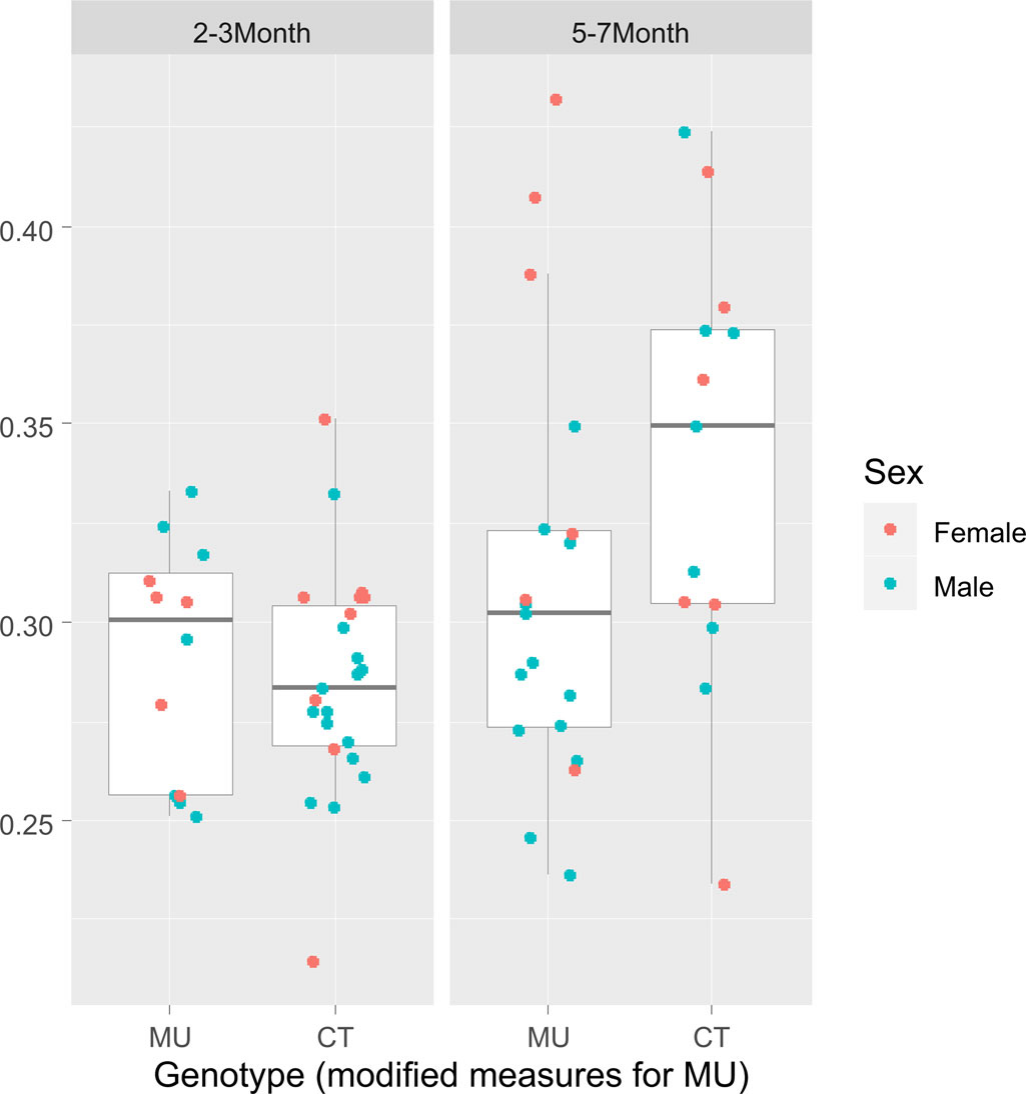
**Prolonging the duration of a subset of respiratory intervals to mimic breathing-swallowing coordination in MU mice increased the CV2 to CT values.** Using the same analytical approach as in the original respiratory sequence measured in MU mice, one would now conclude that the genotype is not statistically significant (*P*=0.31; age remains significant with *P*<0.001). The respiratory sequences of MU mice were modified *in silico* by extending the duration of every tenth interval by 50%, as described in the Materials and Methods section.

The reduced CV2 in MU mice suggests that the difference in the respiratory sequences in MU and CT mice is based on the temporal modulation of a subset of individual respiratory intervals (see also Discussion). In mutant mice, those modulations seem to be less pronounced, resulting in a smaller CV2 value, reflecting reduced intrinsic variability. To address our hypothesis that the difference in CV2 indeed could be explained by modulating the duration of a subset of respiratory intervals, we mimicked in our collected data an extension of the duration of every tenth interval in the respiratory sequence of MU mice by 50%, corresponding to an average of about 75 ms (see Materials and Methods) (Fig. 6). By so doing, rendered the CV2 values of the respiratory sequence of MU mice more comparable to that of CT mice.

## DISCUSSION

The cerebellum is known to be part of the neuronal circuitry activated during respiratory challenges, such as hypercapnia or hypoxia (Macey et al., 2005; Parsons et al., 2001). There has been, however, no clear experimental evidence supporting a role of the cerebellum in spontaneous, unobstructed (eupneic) breathing of normoxic air (Ebert et al., 1995; Moruzzi, 1940; van der Heijden and Zoghbi, 2018; Xu and Frazier, 2002; Xu et al., 1995). Consistent with previous findings, our results show that loss of cerebellar function does not affect the average respiratory rate or the coefficient of variation of eupneic respiration in mice. Analysis of the CV2, however, showed a significantly reduced variability, i.e. a lower CV2 in MU mice compared to their CT littermates (Fig. 4). The CV2 is uniquely sensitive to brief changes in rhythmicity, such as changes of durations of individual intervals as long as these changes occur repeatedly, regularly or irregularly, throughout the interval sequence (Holt et al., 1996). Our findings suggest that loss of cerebellar function is associated with a loss of modulation of select respiratory intervals. We tested this idea *in silico* by artificially prolonging the durations of a subset of respiratory intervals in the respiratory sequences of ataxic mice, thus mimicking a proposed sporadic modulation of respiratory intervals by an intact cerebellum. Increasing the duration of every tenth respiratory interval by 50% increased the CV2 of the MU sequence to values that no longer differed from CV2 values of CT mice (Fig. 5). At the same time, the changes in average rate and CV caused by this manipulation were insignificant.

The biological function of a cerebellar modulation of select respiratory intervals is unknown. However, a possible function could be the proper temporal coordination of breathing with swallowing movements (Hardemark Cedborg et al., 2009). Inappropriate temporal coordination of swallowing with respiration can lead to dysphagia, increasing the risk of aspiration pneumonia (Yagi et al., 2017). Dysphagia is a common symptom in patients with cerebellar disease (Ramio-Torrentia et al., 2006) and a potential anatomical substrate for a cerebellar coordination of brain stem pattern generators controlling breathing and other orofacial movements exists in form of extensive reciprocal connections between the cerebellum and brain stem (Asanuma et al., 1983; Teune et al., 2000; Whiteside and Snider, 1953).

While our findings would be consistent with a proposed cerebellar role in the coordination of breathing with orofacial movements (Bryant et al., 2010; Lu et al., 2013), additional experiments involving simultaneous measurements of respiratory and swallowing movements as well as manipulations of cerebellar function are needed to address this question.

While our data suggest an age-related difference in the CV2 of the respiratory sequence, answers to the question of whether a CV2 increase with age is linked to cerebellar function will require a larger sample size and preferably longitudinal data from the same specimens as they age.

### Conclusions

The cerebellum has long been known to be involved in respiration under conditions when respiration is challenged, such as during hunger for air induced by hypoxia or hypercapnia. Spontaneous, unobstructed (eupneic) breathing, by contrast, did not seem to involve the cerebellum. Here we applied a measure of intrinsic rhythmicity, the CV2 (Holt et al., 1996), which is sensitive to brief sporadic changes in the rhythmicity that are not captured by the CV but are reflected in the CV2. Our results show a reduced CV2 in the respiratory behavior of a mouse model of cerebellar ataxia (White et al., 2014) compared to their healthy littermates. A sporadic modulation of select respiratory intervals could serve the purpose of coordinating respiration with swallowing (Hardemark Cedborg et al., 2009). Whether this task does indeed involve the cerebellum remains to be shown. Coordinating breathing with swallowing, however, constitutes a timing problem, which fits well with the widely accepted role of the cerebellum in timing and temporal coordination of sensorimotor activity (Braitenberg, 1961; Ivry et al., 2002; Mauk and Buonomano, 2004).

## MATERIALS AND METHODS

### Animals

A total of 67 adult mice, 26 females and 41 males, were used in the present study. We compared two groups of mice, one suffering from cerebellar ataxia due to selective loss of Purkinje cell GABAergic synaptic transmission [mutant (MU): *L7^Cre^;Vgat^flox/flox,^*, *n*=31] and their unaffected littermates [control (CT): *Vgat^flox/flox^*, *n*=36] that served as controls (see also Table 1 for details). Transgenic *L7^Cre^* mice (JAX stock# 006207) were from a mixed background as they were originally generated on a Black Swiss background and bred using C57BL/6, and the *Vgat^flox/flox^* knock-in mice (JAX stock # 012897) were also from a mixed background as they were generated using the 129S6 background, chimeras bred to FVB/N or C57BL/6 and eventually bred to C57BL/6J (please refer to the JAX website for full details). Since establishing our own colony, the inter-crossed alleles have been maintained on the C57BL/6J background. Mice were either 2–3 or 5–7 months old at the time of experiments. During breeding, the day a vaginal plug was detected was considered embryonic day 0.5 and the day of birth postnatal day 0. Genotyping was performed with standard PCR reactions that detect the presence of the *Cre* and *flox* alleles (White et al., 2014). Briefly, ear clips were taken from the mice before weaning and then incubated overnight at 60°C in lysis buffer (1.5 mM Tris pH 8.8, 0.5 M EDTA, 10% Tween-20, and double distilled H_2_O) supplemented with proteinase K. The DNA was then processed for PCR using the following primer sequences: *Cre*: Forward: TAA AGA TAT CTC ACG TAC TGA CGG TG, Reverse: TCT CTG ACC AGA GTC ATC CTT AGC; *Flox*: Common: TCC TTT GTG GCT TCC TTC CG, Wild type: GGA TAG AAG AAG TGT GGA CC, Mutant: GCA GTG GAC CTT GGA TGT CTA TC. The experimental protocols were approved by the Institutional Animal Care and Use Committee of The University of Tennessee Health Science Center.

### Measurements of respiratory behavior

Respiratory behavior was monitored for 30 min by placing mice in a plethysmograph consisting of a glass container within which mice could move about freely (Fig. 1). A constant flow (1 l/min) of fresh air was directed into the chamber through an opening in the lid with a second opening in the lid serving as an outlet. While the mice were in the chamber, a box was placed over the chamber, blocking direct light.

The plethysmograph chamber represented a novel environment for the mice, which elicited natural exploratory behavior that includes sniffing, i.e. spontaneous bouts of respiration at frequencies >5 Hz (Cao et al., 2012; Wesson et al., 2011). All analysis of respiratory behavior for the 30 min observation time thus include episodes of sniffing.

Inspiration movements cause the air pressure in the plethysmograph chamber to slightly increase. These pressure changes were measured with a pressure transducer (Validyne Engineering, USA). The voltage output of the transducer reflected pressure increase as a decrease in voltage. A decrease in the raw voltage data (example shown in Fig. 1) thus corresponds to inhale movements, and the minima of voltage troughs correspond to the end-of-inhale movements. Voltage data were digitized at 2 kHz using an analogue to digital converter (CED 1401, Cambridge Electronic Design, UK) and stored on computer hard-disk for off-line analysis using signal processing software (Spike 2, Ver. 7, Cambridge Electronic Design, UK). The minima of each voltage trough in the raw data were detected and the times of their occurrences were used as temporal markers of end-of-inspiration. All data analysis was based on the resulting time series of end-of-inspiration markers.

### Statistical analysis

Further analysis of different aspects of the respiratory rhythm, such as mean interval duration, coefficient of variation (CV=standard deviation/mean), and intrinsic variability (CV2) were based on the end-of-inspiration times. The CV2 provides a measure of the similarity of two adjacent inter-inspiration intervals (Eqn 1):(1)
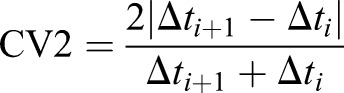
We evaluated the CV2 of each respiratory sequence as the average of all CV2 values calculated for each interval pair, as described in (Holt et al., 1996). All quantitative analyses are based on the average CV2 value. Descriptive boxplots including all animals by genotype as well as age at time of testing and sex are shown in Fig. 2. Linear models were fitted relating the respective outcome measure to genotype while adjusting for age and sex. Model fit was verified by residual plots to verify approximate normality of residuals. The most important conclusions for CV2 were verified by a bootstrap resampling approach. Because respiratory function might be affected by age and/or sex, it is important to adjust for these factors when evaluating systematic differences with respect to genotype. We do this by adding factors for sex and age-group in our regression approach (Kutner et al., 2005). We follow the common approach to exclude a factor if it is not statistically significant (*P*<=0.05) because each additional factor reduces the degrees of freedom in the testing for the genotype-difference, which is our main focus.

### Artificial modulation of respiratory interval sequences

To mimic a proposed cerebellar-dependent extension of the duration of individual respiratory intervals, we artificially increased the durations of 10% of the intervals in the respiration sequences measured in MU mice. Specifically, in each MU respiratory sequence we increased the duration of every tenth respiratory interval by 50% of its original duration, which translated to an average increase of around 75 ms.
